# Exploring website gist through rapid serial visual presentation

**DOI:** 10.1186/s41235-019-0192-1

**Published:** 2019-11-20

**Authors:** Justin W. Owens, Barbara S. Chaparro, Evan M. Palmer

**Affiliations:** 1 Department of Psychology, Wichita State University, Wichita, KS USA; 2 Google, Inc., Mountain View, CA USA; 3 Department of Human Factors and Behavioral Neurobiology, Embry Riddle Aeronautical University, Daytona Beach, FL USA; 4 Department of Psychology, San José State University, San Jose, CA USA

**Keywords:** Web page gist, Website gist, Perceptual gist, Scene perception, Rapid serial visual presentation

## Abstract

**Background:**

Users can make judgments about web pages in a glance. Little research has explored what semantic information can be extracted from a web page within a single fixation or what mental representations users have of web pages, but the scene perception literature provides a framework for understanding how viewers can extract and represent diverse semantic information from scenes in a glance. The purpose of this research was (1) to explore whether semantic information about a web page could be extracted within a single fixation and (2) to explore the effects of size and resolution on extracting this information. Using a rapid serial visual presentation (RSVP) paradigm, Experiment 1 explored whether certain semantic categories of websites (i.e., news, search, shopping, and social networks/blogs) could be detected within a RSVP stream of web page stimuli. Natural scenes, which have been shown to be detectable within a single fixation in the literature, served as a baseline for comparison. Experiment 2 examined the effects of stimulus size and resolution on observers’ ability to detect the presence of website categories using similar methods.

**Results:**

Findings from this research demonstrate that users have conceptual models of websites that allow detection of web pages from a fixation’s worth of stimulus exposure, when provided additional time for processing. For website categories other than search, detection performance decreased significantly when web elements were no longer discernible due to decreases in size and/or resolution. The implications of this research are that website conceptual models rely more on page elements and less on the spatial relationship between these elements.

**Conclusions:**

Participants can detect websites accurately when they were displayed for less than a fixation and when the participants were allowed additional processing time. Subjective comments and stimulus onset asynchrony data suggested that participants likely relied on local features for the detection of website targets for several website categories. This notion was supported when the size and/or resolution of stimuli were decreased to the extent that web elements were indistinguishable. This demonstrates that schemas or conceptualizations of websites provided information sufficient to detect websites from approximately 140 ms of stimulus exposure.

## Significance

In the 25+ years since the advent of the MOSAIC web browser, the web has become an integral part of modern society. Research has demonstrated that people have expectations about website layouts and can judge a web page’s usability, trustworthiness, and visual appeal in a glance. While we understand that users have expectations about websites, and that they can rapidly categorize websites, little is known about the underlying cognitive mechanisms for visually processing websites or forming layout expectations.

Literature on how websites are perceived, attended to, and classified mirror various aspects of the scene perception literature, at least on the surface. Extensive research on scene perception covers topics including scene gist, scene classification, scene processing, and visual search. Due to these shared aspects, scene perception theories and methodologies, specifically those related to scene gist, could be used to explore the cognitive and perceptual processing of websites.

The results of this study demonstrate that website schemas or conceptualizations provided sufficient information to distinguish between different types of websites under rapid serial visual presentation. This suggests quick and efficient website perception may utilize a combination of gist-like and diagnostic feature processing.

## Background

In 1993, the MOSAIC web browser ushered in the internet age, exposing modern culture to web pages, a new form of stimuli. In the 25+ years since, web pages have become integral to society. In 2016, 87% of US adults were online, up from 52% in 2000 (Pew Research, 2013). Given the tremendous contact with this relatively new class of stimuli, we wondered whether people could accurately detect website categories (e.g., news, shopping, search, social media) with exposure durations equivalent to a single glance.

Jahanian, Keshvari, and Rosenholtz (2018) established that participants could accurately categorize web pages with only 120 ms of exposure of the stimulus. These short presentations were sufficient for accurate detection of ads on the web pages and localization of the navigation menu. While participants used web page text as an information source during the task, they still had above chance performance in a classification task when the text was inverted and reflected. Thus, Jahanian et al. (2018) demonstrated that participants can rapidly extract important featural information from a web page within a single glance and accurately categorize it. The present research expands the Jahanian et al. (2018) work by testing participants’ ability to categorize web pages using an RSVP procedure, using different web page categories, more web page stimuli per category, and different stimulus sizes and resolutions.

Web users have well-established expectations for website layout and formatting (Bernard, 2001, 2003; Bernard & Sheshadri, 2004; Di Nocera, Capponi, & Ferlazzo, 2004; Granka, Hembrooke, & Gay, 2006; Owens, Chaparro, & Palmer, 2011; Owens, Palmer, & Chaparro, 2014; Roth, Schmutz, Pauwels, Bargas-Avila, & Opwis, 2010; Shaikh, Chaparro, & Joshi, 2006; Shaikh & Lenz, 2006). For instance, users expect navigation on the left or top of a website, and advertising on the top or right (Bernard, 2001; Bernard & Sheshadri, 2004; Shaikh et al., 2006; Shaikh & Lenz, 2006). Such layout expectations are cross-cultural (Bernard & Sheshadri, 2004; Shaikh et al., 2006), exist for specific types of websites (Roth et al., 2010), and are affected by users’ experience and expertise (Di Nocera et al., 2004; Roth et al., 2010). While users rely on website layout conventions, they can adapt to violations of these conventions, despite the decreased usability (McCarthy, Sasse, & Riegelsberger, 2004; Owens et al., 2014; Santa-Maria & Dyson, 2008; Tzanidou, Petre, Minocha, & Grayson, 2005).

Few studies have investigated what information can be derived from web pages in a single glance. With the exception of Jahanian et al. (2018), previous researchers mainly focused on subjective user impressions, such as visual aesthetics, trustworthiness, and perceived usability (Albert, Gribbons, & Almadas, 2009; Jiang, Wang, Tan, & Yu, 2016; Lindgaard, Dudek, Sen, Sumegi, & Noonan, 2011; Lindgaard, Fernandes, Dudek, & Brown, 2006; Thielsch & Hirschfeld, 2012; Tuch, Presslaber, Stocklin, Opwis, & Bargas-Aliva, 2012). For instance, judgments about web page aesthetics are almost as consistent between 50 ms, 500 ms, and unlimited exposure durations (Lindgaard et al., 2006, 2011). With exposures as low as 17 ms, aesthetic judgements have been shown to correlate with web page prototypicality and visual complexity (Tuch et al., 2012). With only slightly longer display durations (i.e., 50 ms), trust and perceived usability could be reliably rated (Albert et al., 2009; Lindgaard et al., 2011). Such 50-ms exposure durations are substantially shorter than the average fixation duration of 200–250 ms (Rayner, 2009).

The studies reviewed above raise the question: how can these types of judgments occur reliably within a single glance? Additionally, since users seem to have well-defined conventions for websites, how are website layouts perceived and represented cognitively? With little work exploring perceptual and cognitive representations of web pages and how quickly they can be accessed, we explored these questions with well-established methodologies from the scene perception literature.

### Scene perception and gist

A theme common to scene perception literature has been how easily participants recognize visual scenes from very brief exposure durations. In general, global information of a scene is processed first, followed by local information (Navon, 1977). In the case of an outdoor scene, this is analogous to processing the forest before the trees. It seems that observers rely on global information to classify scenes, and work has provided additional detail about the sorts of holistic, global scene information that might be important for recognition.

Several scene perception theories incorporate processing of global information for the recognition of objects within a scene, including the perceptual schema model, the priming model, and contextual guidance model (Friedman, 1979; Henderson & Hollingworth, 1999; Oliva & Torralba, 2007; Torralba, Oliva, Castelhano, & Henderson, 2006). Such holistic representations have also been integrated into other vision theories, such as newer versions of Wolfe’s Guided Search model (Wolfe, Võ, Evans, & Greene, 2011) and the spatial envelope theory (Oliva & Torralba, 2001).

Changes to global statistics can affect scene perception (Joubert, Rousselet, Fabre-Thorpe, & Fize, 2009). Some research has suggested that superordinate categories (Rosch, 1978) have less bias than basic categories (Loschky & Larson, 2010). When scenes share global properties or lack distinct global properties, correctly distinguishing between scenes becomes more difficult (Greene & Oliva, 2009b; Loschky & Larson, 2010).

Observers’ ability to rapidly extract the “gist” of a scene has also been researched extensively. Seminal gist research found that participants detected rapidly presented target scenes above chance after being prompted with just a verbal description or image (Potter, 1975, 1976). Research describes scene gist as the extracted meaning of a scene occurring within a single fixation, possibly with little-to-no attention, based on global processing of visual information (Fei-Fei, Iyer, Koch, & Perona, 2007; Fei-Fei, VanRullen, Koch, & Perona, 2002; Greene & Oliva, 2009a, 2009b; Intraub, 1980, 1981; Larson & Loschky, 2009; Oliva, 2005; Potter, 1975, 1976). Information contained within scene gist may consist of a semantic label, a limited number of objects, and the spatial layout of objects (Oliva & Torralba, 2006).

Participants performed better when they were prompted with pictures than with text descriptors (Potter, 1976). Moreover, when prompts have more information, (i.e., butterfly vs animal), performance typically increases (Intraub, 1981). Longer displays of scenes also result in richer descriptions of the scenes or features detected (Fei-Fei et al., 2007; Intraub, 1981; Loftus, Nelson, & Kallman, 1983). Fei-Fei et al. (2007) found a rich variety of information, including object identities and scene classifications, could be derived from a scene in 107 ms. Other research has shown that objects can be recognized as quickly as 100 ms (Liu, Agam, Madsen, & Kreiman, 2009). In fact, gist can be extracted in the absence of fine detail, from degraded scenes, when objects are difficult to process (Larson & Loschky, 2009; Meng & Potter, 2008; Oliva & Torralba, 2007; Potter, 1975, 1976; Rousselet, Joubert, & Fabre-Thorpe, 2005; Torralba, 2009), or even if multiple scenes are presented simultaneously (Potter & Fox, 2009).

Oliva (2005) proposed that gist occurs in conceptual and perceptual forms. Perceptual gist represents the depiction of the scene defined by its global features, while conceptual gist is the scene’s semantic meaning extracted during the cognitive processing that occurs after viewing the scene. Perceptual gist influences conceptual gist.

Extraction of semantic information from scenes is robust, even with visually degraded scenes. For instance, scenes can be detected and recognized even when they are partially occluded (Meng & Potter, 2008), inverted (Diamond & Carey, 1986; Epstein, Higgins, Parker, Aguirre, & Cooperman, 2006; Evans & Treisman, 2005; Harding & Bloj, 2010; Kelley, Chun, & Chua, 2003; Meng & Potter, 2008; Shore & Klein, 2000), have had color removed (Meng & Potter, 2008; Rousselet et al., 2005), contain object inconsistencies (Biederman, Mezzanotte, & Rabinowitz, 1982; Davenport, 2007; Davenport & Potter, 2004), and even when the scenes are low resolution or poor quality (Loschky, Hansen, Sethi, & Pydimarri, 2010; Oliva & Schyns, 1997; Torralba, 2009). The recognition and detection of scenes in such scenarios has been attributed to the semantic information derived from the scene.

#### Scene gist methods

During studies exploring scene gist, viewers typically see a prompt, followed by a scene stimulus for up to a few hundred milliseconds, and then a masking stimulus. Masks stop perceptual processing of a stimulus, allowing for more accurate estimates of requisite scene processing time (Potter, 1976). Another approach has been to display a prompt followed by an RSVP stream of scenes instead of single scene and mask. The rapid presentation of stimuli, one after the next, effectively halts perceptual and conceptual processing of the previously presented stimuli (Intraub, 1984; Potter, 1976). Following the display sequence, viewers are asked whether any stimulus in the stream matched the prompt shown at the beginning of the task. With either approach, stimuli are often displayed from 10 to several hundred milliseconds and participants typically achieve above chance performance detecting targets provided by the prompt.

Detecting gist requires exposure durations shorter than a single fixation (Potter, 1975, 1976). This can be accomplished using sufficiently short display durations with appropriate inter-stimulus intervals (ISIs), which remove stimuli from the screen without masking and allow for continued processing. Loftus, Shimamura, and Johnson (1985) noted that performance using unmasked stimuli was equivalent to approximately 100 ms of additional exposure time, due to the prolonged sensory presence of the stimuli in the visual icon (Neisser, 1967; Sperling, 1960). Potter and Fox (2009) found that participants readily detected targets regardless of whether RSVP streams incorporated ISIs, but demonstrated that when ISIs were present, performance was relatively worse. During recognition tasks, participants performed similarly regardless of whether RSVP streams incorporated ISIs.

### Website categories

Web pages are complex documents, consisting of a variety of elements arranged spatially within a single page. Some previous classification attempts have relied on groups of elements or the type of elements found within a web page, sometimes in combination with previous personal experiences (Crowston & Williams, 2000; Dillon & Gushrowski, 2000), while other attempts have focused on automation and examining the hierarchy and the occurrences of types of text within a web page (Rehm, 2002; Santini, 2006). These methods have typically resulted in researchers or automation creating genres, but not users. Jahanian et al. (2018) developed ten web page categories for their study: art place, blog, company, computer game, helpline, news, online tutorials, shopping, society, and tourism. The authors derived categories based on considerations of web page use, which were validated in a pilot study.

The web evolves over time, which has interesting implications for classification schemes. Santini (2007) noted that some types of websites may emerge or may just be unknown. For instance, before blogs were a mainstay on the Internet, they were considered an emerging genre. Similarly, Crowston and Williams (2000) found a large portion of their genres as being previously unknown. Both sets of authors argued that web pages may be classified into multiple genres. For example, market research from NM Incite found that of the largest social networking websites, three were actually blogs (Nielsen, 2012), which included Blogger, WordPress, and Tumblr. In one study, participants examined United States’ individual state website home pages by placing them into groups, and then examining them in terms of form, function, and content over time (Ryan, Field, & Olfman, 2002). The importance of these dimensions shifted over time. However, none of these classification methods address whether websites can be classified into a similar taxonomy as scenes.

### Web page gist

For gist processing of websites to occur, they would need to have characteristic spatial structure that the human visual system could learn and harness for rapid categorization, as in scenes. As reviewed above, web pages can be categorized into genres, and may evolve over time (Crowston & Williams, 2000; Santini, 2007). Web designs tend to follow a certain structure, often with navigation regions on the left side and top of the page, content in the middle, and advertising regions on the right (Bernard, 2001, 2003; Bernard & Sheshadri, 2004; Di Nocera et al., 2004; Granka et al., 2006; Owens et al., 2011, 2014; Roth et al., 2010; Shaikh et al., 2006; Shaikh & Lenz, 2006). People expect web pages to follow these layout conventions and may react negatively when the conventions are violated (Owens et al., 2014). People exhibit a phenomenon called “banner blindness” where they will ignore areas of websites where ads are most expected, even if they know that relevant information may be located there (Benway, 1998; Owens et al., 2011, 2014). This suggests that habitual ignoring of web page regions may be based on the spatial structure of the website, rather than the visual characteristics of the elements.

Thus, it seems that there is evidence that people develop gist-like representations of web pages (Jahanian et al., 2018). We believe the human visual system is tuned to statistical regularities in the world and exploits those regularities to guide behavior whenever possible (e.g., Turk-Browne, Jungé, & Scholl, 2005). To further determine whether there is indeed gist processing of web pages at a glance, we employed an RSVP paradigm, as described in the present study’s experiments.

### The current work

Given that humans can recognize scenes in a glance, we wondered whether they could recognize different types of websites in a glance. To investigate these issues, we first had participants classify website screenshots into multiple categories as part of a pilot study. A sample of 271 participants recruited from Wichita State University and Mechanical Turk classified 132 de-branded websites into one of nine categories: news, search, shopping, social networks, blogs, maps, corporate, general knowledge websites, or none. Social networking web pages were classified as both blogs and social networks, so they were combined into a single blogs/social networks category. Web pages with over 80% agreement for a single category, but no more than 20% agreement for a second category, were selected for the study (see Table 1 for category agreement results). These categories had websites that participants would likely have experience using, but also represent some of the oldest or largest website categories found on the Internet.

**Table 1:** Participant Agreement For Website Classification

Website category	N	Overall rank	Agreement (*SD*)
Search websites	276	3	95.43% (4.75%)
Shopping websites	276	1	97.95% (3.33%)
Social networks/blogs	276	3	94.95% (5.16%)
News websites	276	3	94.81% (4.33%)

After determining website categories, we tested users’ ability to detect a specific type of website within an RSVP stream of other websites in Experiment 1. In Experiment 2, we explored the effects of size and blur on observers’ ability to rapidly detect websites in an RSVP stream.

## Experiment 1

Experiment 1 was conducted to determine whether specific web pages could be detected with above chance accuracy during an RSVP task, when displayed for less than a fixation (≤ 140 ms). Comparisons were provided by having participants detect upright and inverted natural scenes in separate conditions. Upright natural scenes provided a best case comparison for gist perception and scene-related performance, while inverted natural scenes provided a degraded performance comparison by interrupting the extraction of a scene’s semantic meaning.

Inverted scenes have several advantages. Features such as spatial structure, color, and luminance remain consistent regardless of orientation, but the change in orientation tends to interfere with perception of semantic scene categories. Using inversion (180-degree rotation) as a method of degrading scenes has had mixed results. Inversion reduces detection of scene targets during RSVP tasks (Evans & Treisman, 2005), detection of changes in a scene (Kelley et al., 2003; Shore & Klein, 2000), but not detection of animals and humans (Rousselet et al., 2003). Inversion has been shown to reduce performance when used in combination with occlusion (Meng & Potter, 2008) and changes in luminance (Harding & Bloj, 2010), but not significantly when combined with gray scaling (Nandakumar & Malik, 2009) and jumbling scenes (Zimmermann, Schnier, & Lappe, 2010).

In this study, a staircase procedure was used to estimate stimulus onset asynchrony (SOA) durations necessary for several levels of performance for upright scenes, inverted scenes, and web page targets in an RSVP task. SOAs represent the amount of time elapsed between the onset of two stimuli. We developed three hypotheses:

- **H**_**1**_**:** Participants will be able to discriminate between categories for both scenes and web pages based on stimulus exposures lasting less than a fixation, but participants will have worse performance for web pages than for scenes. We felt that web page perception would be worse because participants have seen more scenes than web pages during their lifetime.
- **H**_**2**_**:** The necessary performance to reach desired accuracy thresholds will be lower for categories with higher agreement.
- **H**_**3**_**:** Performance will be better for upright scenes than inverted scenes, but inverted scenes will be similar or better than web page-related SOAs. Similarly to H_1_, we felt that participants would have more experience with scenes as a whole during their lifetime.

### Methods

#### Participants

Twenty-two college students from Wichita State University participated for course credit. All participants provided informed consent and the study was approved by the Wichita State University Institutional Review Board. Two participants did not complete the study and another was omitted from analysis due to poor overall performance (z = − 2.41, M = 865 ms). Of the remaining 19 participants (*M* = 21.16 years, *SD* = 3.67 years; 7 males, 12 females), four self-reported they use the Internet 1–10 h per week, while 15 self-reported using the Internet at least 11 h or more per week. The most common self-reported reasons for using the Internet included e-mail, entertainment, education, and social networking.

#### Apparatus

Participants viewed stimuli on a 22-in. CRT monitor with an 85 Hz refresh rate and 1400 × 1050 pixel (px) resolution, paired with a 2GHz Apple Mac Pro computer running Matlab and RSVP software using PsychToolbox (Brainard, 1997; Kleiner, Brainard, & Pelli, 2007; Pelli, 1997) and QUEST (Pelli, 1987; Watson & Pelli, 1983).

#### Materials

**Fig. 1:**
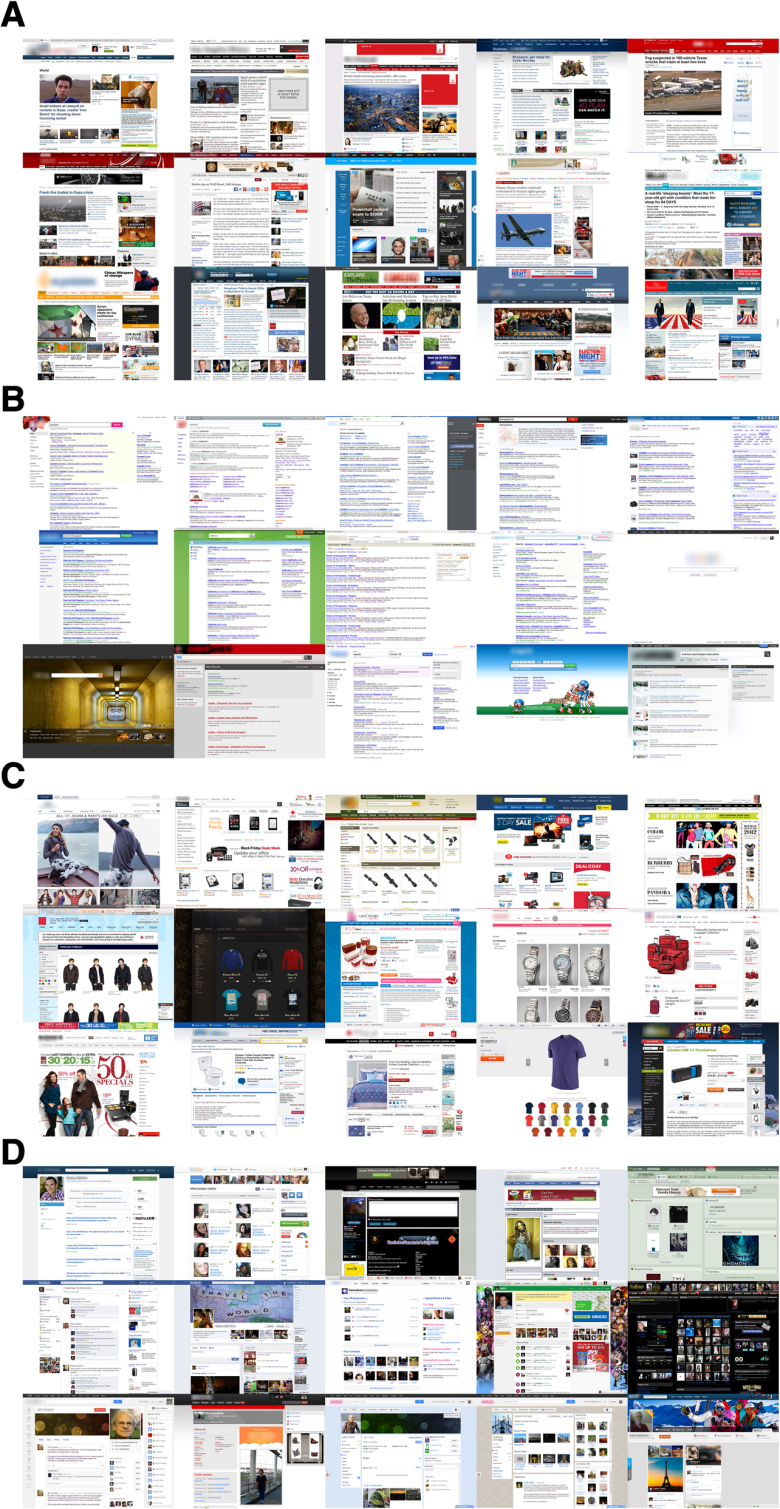
Examples of the website stimuli used in Experiment 1. **a** News. **b** Search. **c** Shopping. **d** Social networking/blogs

Websites and visual scenes were selected as stimuli for the study. All visual stimuli were presented at 512 × 386 px, which subtended 13.69° by 10.34° at a 60 cm distance. As described above, website stimuli were selected from a pilot study in which participants classified screenshots of web pages (in the same resolution as presented in the current study) into one or more categories, yielding 276 screenshots per category (the entire website stimulus set can be downloaded from https://scholarworks.sjsu.edu/psych_pub/28/). Each of the website categories selected for this study had classification agreement scores of above 94%. Due to a configuration error, only 172 screenshots for the social networks/blogs category were used in Experiment 1. See Fig. 1 for examples of the website stimuli.

**Fig. 2:**
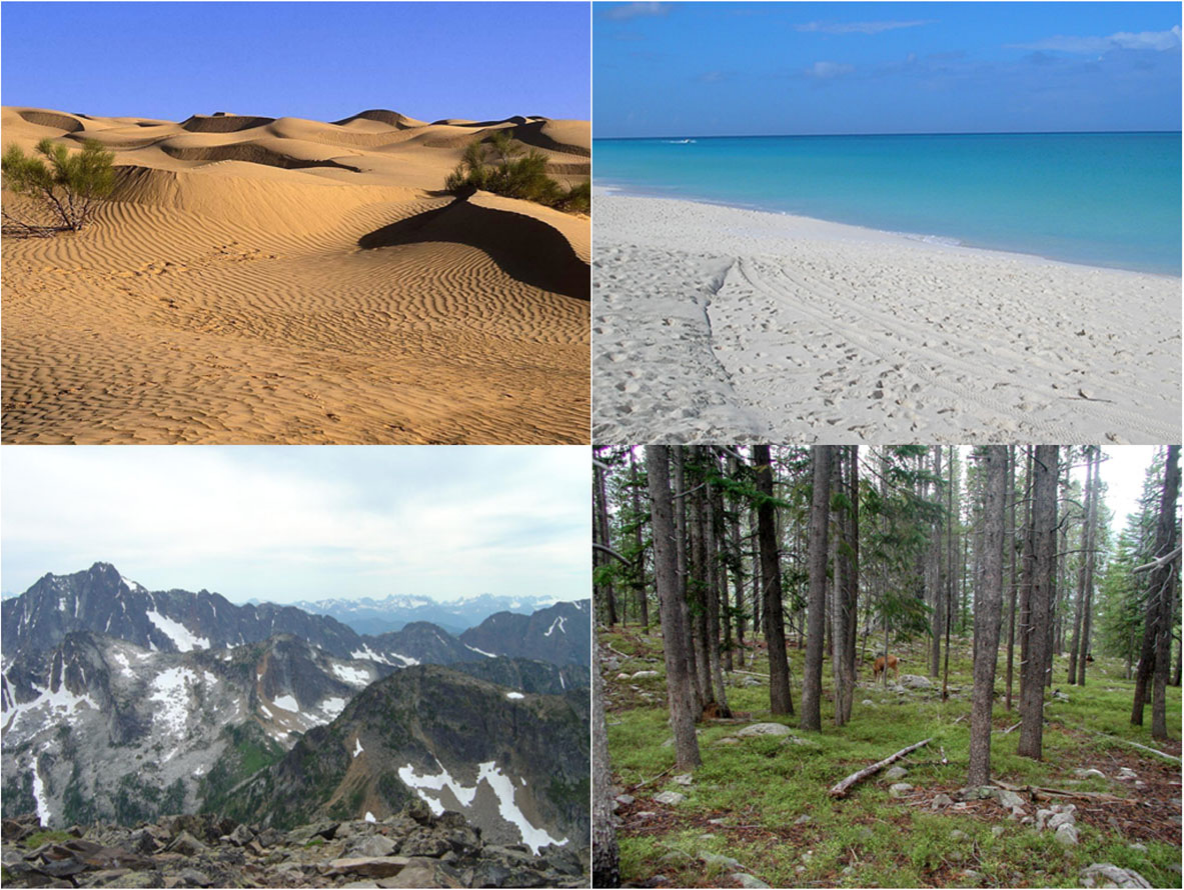
Examples of natural scene stimuli used in Experiment 1. Clockwise from *upper left*: desert, beach, forest, mountains

For the natural scene stimuli, four categories were selected: beaches, mountains, deserts, and forests. For target and distractor categories, 268 and 284 scenes were selected, respectively. Stimuli were downloaded from the SUN database (Xiao, Hays, Ehinger, Oliva, & Torralba, 2010) and Google Image Search. The scene stimuli were validated through pilot testing. Inverted versions of the natural scenes were also created. See Fig. 2 for examples of outdoor scenes.

#### Procedure

Participants were screened for normal color vision and normal or corrected-to-normal visual acuity. The researcher described the experiment procedure and provided descriptions of the stimuli. Participants then were seated at a chinrest where the RSVP program provided instructions on the task and how to respond using the keyboard.

**Fig. 3:**
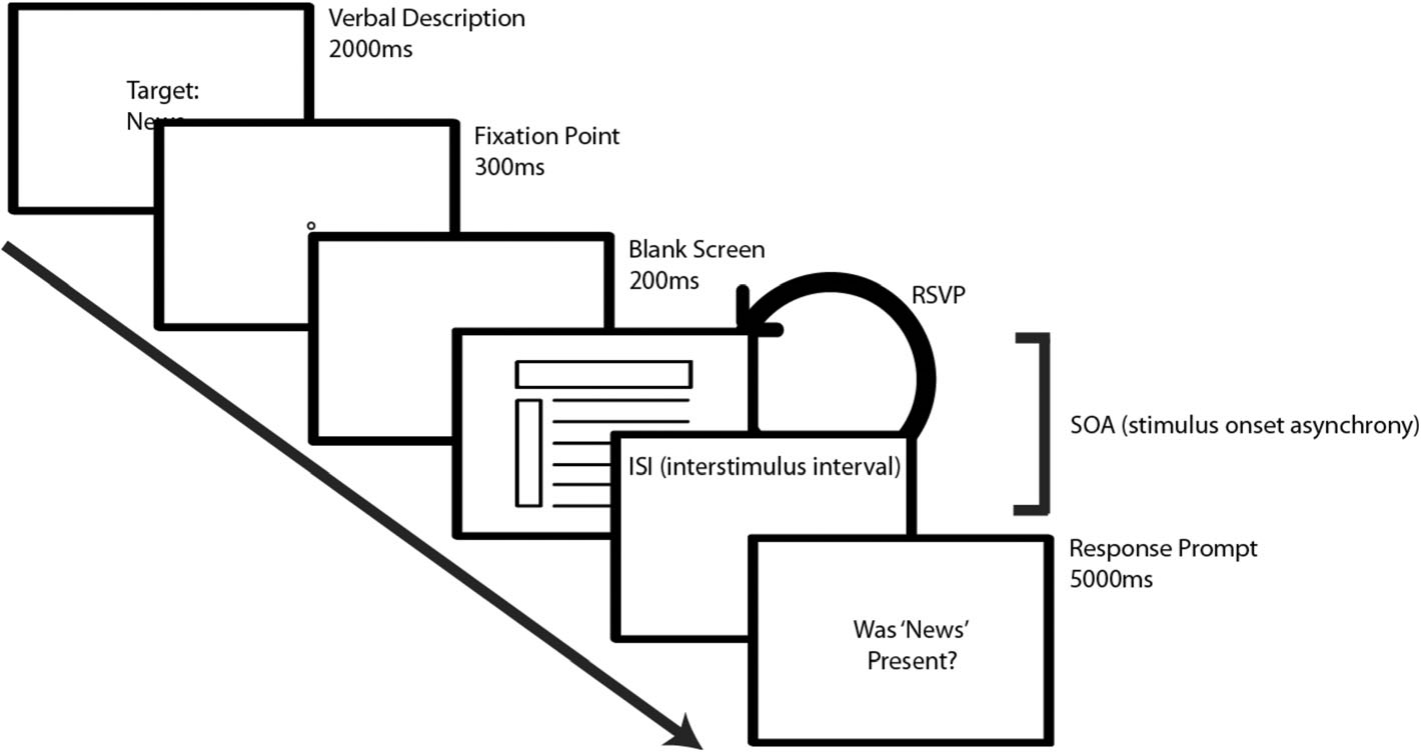
RSVP trial schematic for Experiment 1

The experiment consisted of multiple RSVP trials. Each trial consisted of a brief written description of the target, followed by a fixation point, blank screen, multiple stimuli presented in succession, and a prompt inquiring whether the target category was present in the RSVP stream. See Fig. 3 for a schematic of the trial.

For each website or natural scene category, QUEST statistical-based adaptive staircases (Watson & Pelli, 1983) were initialized for three accuracy thresholds indicating above chance performance (e.g., 60%, 75%, and 90%). Participants completed two practice and 40 experimental trials for each category/threshold condition, equating to 24 practice and 480 experimental trials in total. Half of these trials were target present, while the other half were target absent. The trial order was randomized.

Each trial consisted of 15 randomly selected stimuli from three nontarget categories. Nontarget stimuli had an equal chance of being seen multiple times during the study, but targets were only seen once. In each trial, nontargets were selected without replacement, but between trials, nontargets were selected with replacement. During a target-present trial, a single stimulus from the target category was selected without replacement and placed randomly in the RSVP stream between, but not including, the first and last positions.

For each RSVP trial, the SOA was calculated using the QUEST algorithm. Each QUEST staircase was initialized with several parameters, including the minimum SOA, the maximum SOA, mean, and standard deviation. The minimum SOA was set to one screen refresh, the maximum SOA to one second, the mean set as the median of the range (505.9 ms), and the standard deviation was set as one second.

Each stimulus was displayed for 140 ms or less during the RSVP stream. If the requested SOA exceeded this presentation duration, an ISI followed the stimulus presentation to make up the rest of the time (e.g., a 600-ms SOA would be 140 ms exposure plus 460 ms ISI). A participant’s progression through a staircase was halted when the display duration requested by the QUEST algorithm exceeded 976.5 ms (one second minus two screen refreshes) on ten consecutive trials. The number of trials remaining in the halted staircase were considered a measure of poor performance for that condition.

### Results

#### QUEST mean estimates of SOAs

**Fig. 4:**
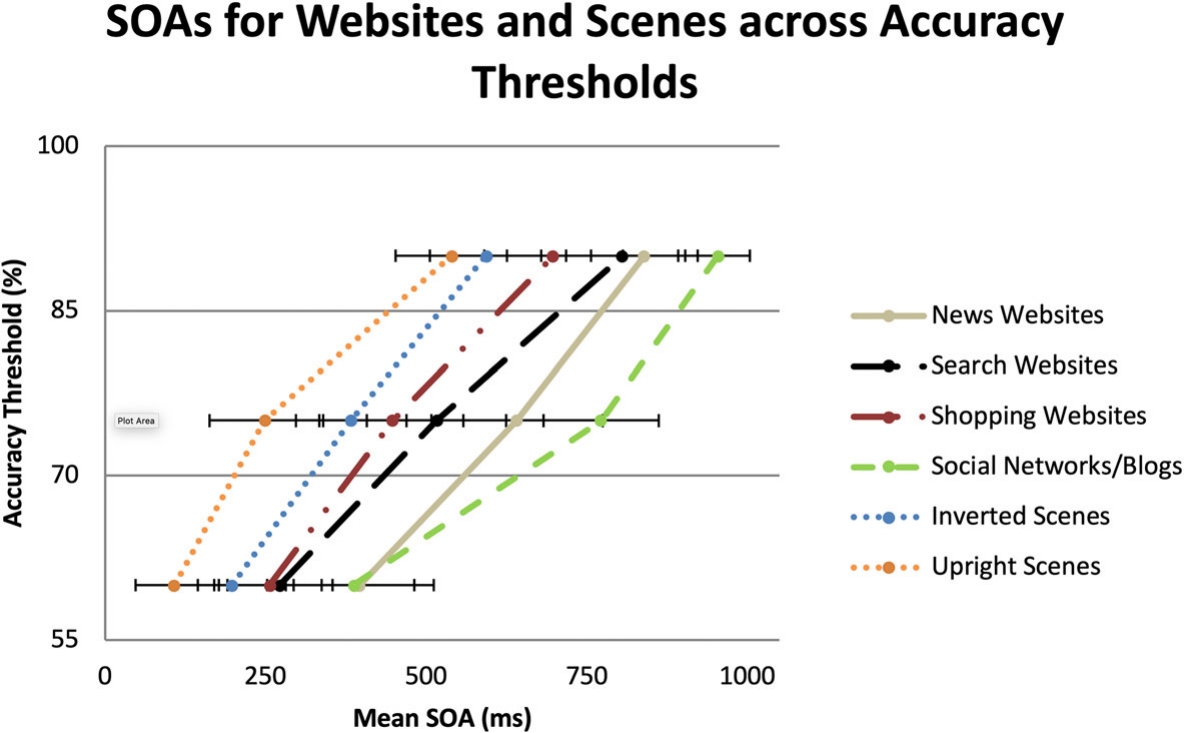
Mean SOAs for websites and scene category accuracy thresholds. Maximum stimulus durations were limited to 140 ms, with blank ISIs making up the rest of the time for each SOA. *Error bars* are within-subjects 95% confidence intervals (Cousineau, 2005; Morey, 2008)

The QUEST mean estimates of SOAs for the 60%, 75%, and 90% accuracy thresholds per each type of web and scene stimuli were calculated. The SOA for each category and accuracy threshold was the presentation time (plus ISI, if applicable) required to detect a stimulus matching a target category during RSVP tasks. The mean SOAs for stimuli types (upright scenes, inverted scenes, and website) are shown in Fig. 4.

#### Halted staircases

The number of trials remaining in a condition in which the staircase was halted was a good indicator of poor performance. Primarily, the QUEST staircase halted most commonly on news websites, social networks/blogs, search websites, and shopping websites at the 90% accuracy threshold, while few participants had halted staircases in the scene conditions. One participant had a halted staircase in the 75% upright mountain conditions and two participants had halted staircases in the 90% inverted mountain condition.

These data points equated to poor performance during the experiment and were not treated as outliers since removal of these data points would artificially lower the SOA for those respective categories and thresholds. Given this, the 90% threshold means for website categories were treated as a lower limit of a respective mean SOA in this study.

#### Overall performance across stimuli types

To establish a high-level overview of how participants performed, the SOAs for stimuli type were collapsed across each respective category for each accuracy threshold (60%, 75%, and 90%). See Table 2 for QUEST mean estimates of SOAs for each stimuli type.

**Table 2:** Mean SOAs for each collapsed stimulus type across accuracy thresholds

Stimuli category	QUEST mean estimates of SOAs in milliseconds (*SD*)			
	60%	75%	90%	Overall
Websites	328 (182)	594 (232)	824 (143)	582 (168)
Upright scenes	109 (28)	248 (190)	539 (202)	299 (123)
Inverted scenes	198 (128)	383 (243)	593 (212)	391 (170)

**Fig. 5:**
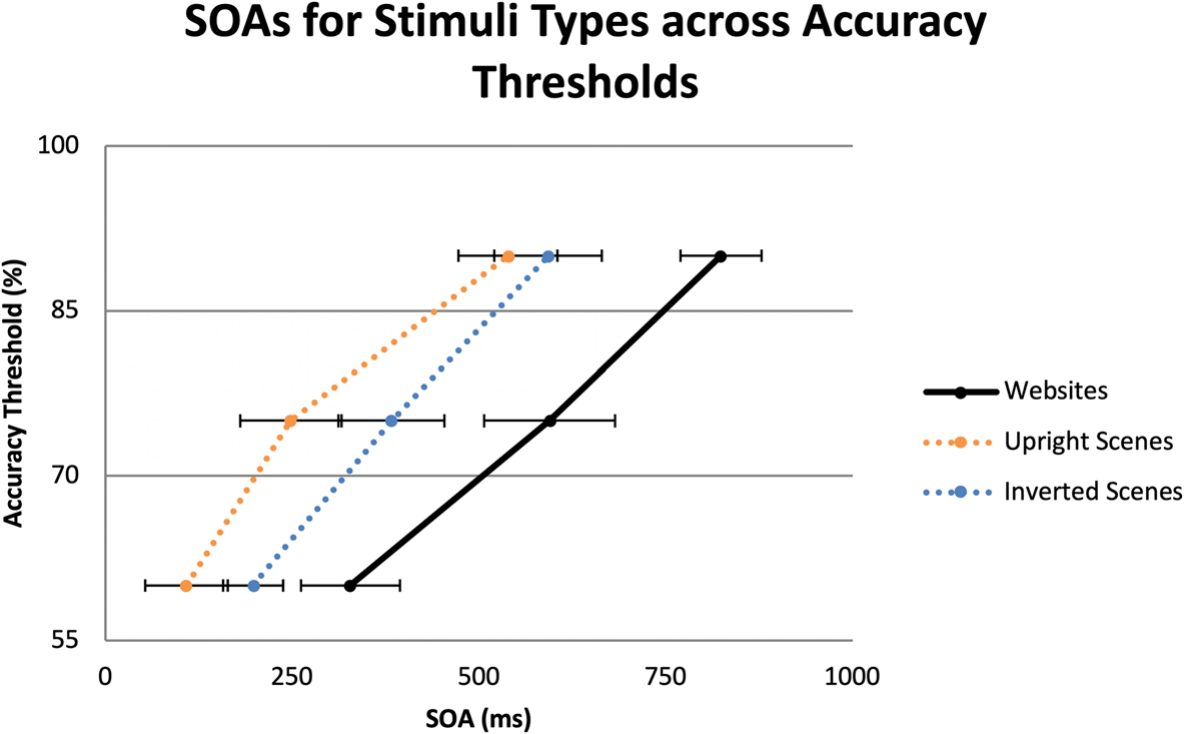
Mean SOAs for scene categories across accuracy thresholds. Maximum stimulus durations were limited to 140 ms, with blank ISIs making up the rest of the time for each SOA. *Error bars* are within-subjects 95% confidence intervals (Cousineau, 2005; Morey, 2008)

A 3 × 3 repeated-measures analysis of variance (ANOVA) was conducted on log transformed SOAs across the stimuli and the accuracy thresholds. The results indicated a main effect for stimuli, *F*(2,36) = 38.32, *p* < 0.01, partial *η*^2^ = 0.68, a main effect for accuracy threshold, *F*(2,36) = 197.05, *p* < 0.01, partial *η*^2^ = 0.92, and an interaction between stimuli type and accuracy threshold, *F*(4,72) = 3.92, *p* < 0.01, partial *η*^2^ = 0.18. Planned pairwise comparisons were conducted to determine which conditions differed. For each accuracy threshold, upright scene and inverted scene SOAs were shorter than the SOA for websites. However, the SOAs for upright scenes and inverted scenes only differed at the 60% and 75% accuracy thresholds, but not for the 90% threshold. Across the three types of stimuli, increases in accuracy threshold resulted in significant increases in SOAs in order to discriminate targets, *p* < 0.01. Figure 5 shows the mean SOAs of the three stimuli types.

At a high-level overview, participants required longer SOAs to detect website targets than upright or inverted natural scenes (283 ms longer for upright and 191 ms longer for inverted scenes). SOAs were shorter for upright scenes than for inverted scenes at 60% and 75% accuracy thresholds, but not at the 90% thresholds. Overall, with only 140 ms of stimulus exposure, participants were able to detect websites in a glance, though they required additional processing time compared to the inverted scene condition, regardless of accuracy threshold.

#### Website category performance

**Fig. 6:**
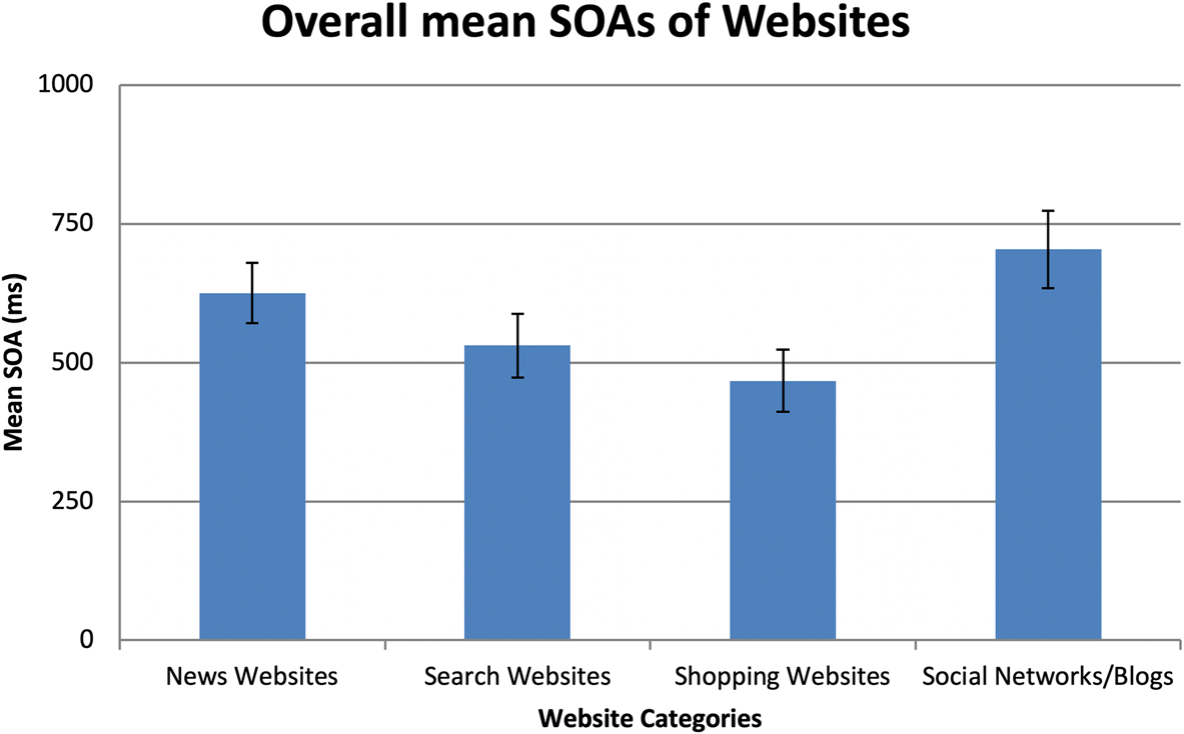
Mean SOAs across the four categories of websites. *Error bars* are within-subjects 95% confidence intervals (Cousineau, 2005; Morey, 2008)

To better understand how performance changed across accuracy thresholds for websites, the individual categories were compared. Results of a repeated measures ANOVA indicated significant differences between the website categories, *F*(3,54) = 12.20, *p* < 0.01, partial *η*^2^ = 0.40. Planned comparisons showed that SOAs associated with shopping and search websites were significantly shorter than SOAs for social networks/blogs. Similarly, the mean SOA for shopping websites was significantly shorter than the SOA for news websites. No significant differences were noted between news websites and social networks/blogs. See Fig. 6 for comparison of mean SOAs across the four categories of website stimuli.

Website categories were analyzed by accuracy threshold to determine how the SOAs differed. Friedman tests revealed that website SOAs were similar to each other at the 60% accuracy level, *Χ*^*2*^(3) = 7.67, *p* = 0.05, *W* = 0.14. However, at 75% accuracy, *Χ*^*2*^(3) = 9.38, *p* = 0.03, *W* = 0.17, and 90% accuracy, *Χ*^*2*^(3) = 12.47, *p* = 0.01, *W* = 0.22, SOAs significantly differed from each other. Planned Wilcoxon tests indicated that social networks/blogs required significantly longer SOAs than both search and shopping websites to obtain 75% and 90% accuracy. While there was a cumulative difference in SOAs between search and news websites, this effect was not significant for any accuracy thresholds. As accuracy thresholds increased, the SOAs for news websites became significantly shorter than the SOAs for social networks/blogs, reaching significance at the 90% accuracy threshold. No differences between search and news websites or shopping and search websites were noted.

### Discussion

The purpose of this study was to compare recognition performance in an RSVP task for upright and inverted scenes with four types of websites (news, shopping, social networking/blogs, and search) presented for less time than a single glance.

#### Natural scenes

Participants were able to detect upright scene targets with above chance accuracy during an RSVP task. At 60% accuracy, SOAs were similar to Potter (1975, 1976) while 75% accuracy SOAs were more similar to those found by Intraub (1981). Performance at 90% accuracy was similar to the findings of Potter and Fox (2009). In summary, our results for upright scenes align with previous results in the literature and suggest that gist was extracted in that condition.

For inverted scenes, 60% accuracy required 89 ms longer for detection than for upright scenes, and 75% accuracy required an additional 135 ms. At 90% accuracy, SOAs for inverted scenes required only 54 ms of additional processing. The inversion of scenes resulted in significant decreases in the gist of a scene, especially at the 60% and 75% thresholds. Because the visual quality of the scene was not modified and confounds were not introduced, it seems that inversion decreased quality of conceptual gist. Thus, upright scenes enabled gist extraction and inverted scenes represented a reduced gist scenario, allowing a comparison with the website conditions to determine the degree of gist extraction.

#### Website stimuli

The mean SOAs of website categories at each accuracy threshold exceeded those for upright and inverted scenes, indicating that participants were able to detect websites within a single fixation, but they required additional processing time to make accurate decisions. The definition of gist typically states that it is not dependent upon the processing of local objects and that it involves the extraction of semantic information from stimulus exposures lasting no longer than a single fixation. While it is possible that gist was extracted from websites given that participants had above chance accuracy from exposures shorter than a single fixation, it is also possible that detection was partially dependent on local web elements.

For both shopping and search websites, gist extraction similar to that of inverted scenes may have occurred at the 60% accuracy threshold, since shopping and search websites did not differ significantly from SOAs for inverted scenes. At the two higher accuracy thresholds, shopping and search websites had longer SOAs than inverted scene performance, though not significantly due to Holm-Bonferroni corrections. For shopping websites, the mean SOA at the 60% threshold did not differ from the SOA of the upright scene condition. Taken together, gist may have contributed to the detection of shopping and search websites.

It seems that the shopping and search website categories were distinctive enough from news websites and social networks/blogs to enable easier detection. While they may have been distinctive in terms of their gist, processing of only a few local features or web elements could also have been sufficient for detecting shopping and search websites but not news or social networking/blogs. The observed SOAs for the website stimuli were long enough for the processing of object features at any accuracy threshold (e.g., Kirchner & Thorpe, 2006). The pictures on shopping websites were primarily merchandise, while most of the search website stimuli lacked pictures. The social media/blogs and news categories both had a mix of text and pictures, perhaps making them less distinguishable in terms of their local elements. In both of these cases, detection of local features, or lack thereof, may have aided detection.

#### Study limitations

Due to a configuration file error, Facebook website stimuli were excluded from social media category targets, though the probability of a single Facebook stimulus being selected as target was 0.36%. The impact of this was examined in the subsequent study, which found no performance differences detecting social networks/blogs when compared to the other three categories.

### Conclusions

This study demonstrated that participants were capable of detecting websites after receiving a category prompt with above chance accuracy from stimulus exposures of 140 ms or less in an RSVP task, but doing so required significantly longer SOAs than for upright and inverted natural scene detection. This suggests that additional processing time, and in some cases additional stimulus exposure, was needed to facilitate the detection.

## Experiment 2

Given that users are able to recognize website categories at a glance, though they required a few hundred ms longer to process the stimuli, we wondered how the size and resolution of website stimuli might affect performance. There are both basic and applied science reasons for looking at website recognition performance under various sizes and resolutions. In terms of basic science, Tuch et al. (2012) suggested that ultra-rapid aesthetic and trustworthiness judgments of websites are based on the low spatial frequency information available in those stimuli. Furthermore, proposed holistic representations such as the spatial envelope in the scene recognition literature suggest that rapid classifications can be made on the basis of low frequency stimulus energy information. Indeed, Torralba (2009) showed that small thumbnail images, measuring just 32 × 32 pixels, were sufficient for participants to identify the semantic category of real-world scenes. Therefore, if participants are rapidly recognizing website stimuli on the basis of information carried in the low spatial frequency channels, using low resolution stimuli should not dramatically affect performance since most of the holistic, spatial-envelope type information would be preserved.

In terms of applied research questions, many desktop and mobile web browsers use small thumbnail representations of web pages in their interfaces. Just how quickly and accurately can people recognize the category membership of those websites from a small, low resolution image? Previous research has shown that small resolution screens may be particularly problematic for displaying websites (Chittaro, 2006). Users have limited viewing of websites and must scroll and zoom to see the entire website in detail. Other research has shown that thumbnails of websites displayed on small device screens were beneficial when the website layouts were preserved, but participants commonly commented about poorly rendered text and indistinguishable images (Lam & Baudisch, 2005). Moreover, they found that participants liked having access to the original layouts, but the layouts themselves were not adequate for finding desired content in some cases.

Screen image size and quality have been investigated in video learning and shown to affect the amount of information acquired. Maniar, Bennett, Hand, and Allan (2008) showed that the amount of information learned from a video was diminished by smaller screen sizes (1.65″) versus larger screen sizes: 2.28″ and 3.78″. Other research has shown that image size and resolution affect the acceptance of video feeds, with common complaints focusing on the inability to discern detail or read text from low bandwidth video (Knoche, McCarthy, & Sasse, 2005).

The purpose of this experiment was to determine the influences of the local features, such as pictures, and how size and resolution influence detection of websites, which is applicable to how websites are commonly displayed on these different types and sizes of displays. From previous literature and the findings of Experiment 1, several hypotheses can be formed:

- **H**_**1**_**:** Participants’ ability to discriminate between categories of websites will be moderated by their size and resolution. Participants’ ability to discriminate between categories will decrease as both size and resolution of the websites decreases. When resolution can be downsampled in terms of image size, the effects of resolution should be greater in smaller stimulus sizes than larger sizes.
- **H**_**2**_**:** Discriminability at smaller sizes will be better for website categories with higher agreement.
- **H**_**3**_**:** Discriminability at lower resolutions will be better for website categories with higher agreement.

### Methods

#### Participants

Twenty-four college students from Wichita State University with normal or corrected-to-normal visual acuity and normal color vision participated in the experiment for course credit. All participants provided informed consent and the study was approved by the Wichita State University Institutional Review Board. Three participants were omitted from analysis due to significantly high error response rates or the inability to follow directions. The remaining 21 participants (*M* = 22.00 years, *SD* = 3.33 years; 5 males, 16 females) all self-reported using the Internet. Seven users reported using the Internet 1–10 h per week. Fourteen users reported using the Internet at least 11 or more hours per week. The Internet was most commonly used for email, entertainment, education, and social networking.

#### Apparatus

The same computer equipment, chinrest, and programming environment that were used in Experiment 1 was used in Experiment 2, except there were changes to the RSVP software for size and resolution conditions.

#### Design

The four website categories from Experiment 1 were used in this experiment and were tested in three different sizes and resolutions each, forming a 4 (website category: news, search, shopping, social networks/blogs) × 3 (size: small, medium, large) × 3 (resolution: low, moderate, high) design. SOAs for the RSVP tasks were set to the 75% accuracy threshold results from the previous experiment: 641.2 ms for news websites, 515.9 ms for search websites, 448.5 ms for shopping websites, and 772.2 ms for social networks/blogs.

#### Stimuli

**Fig. 7:**
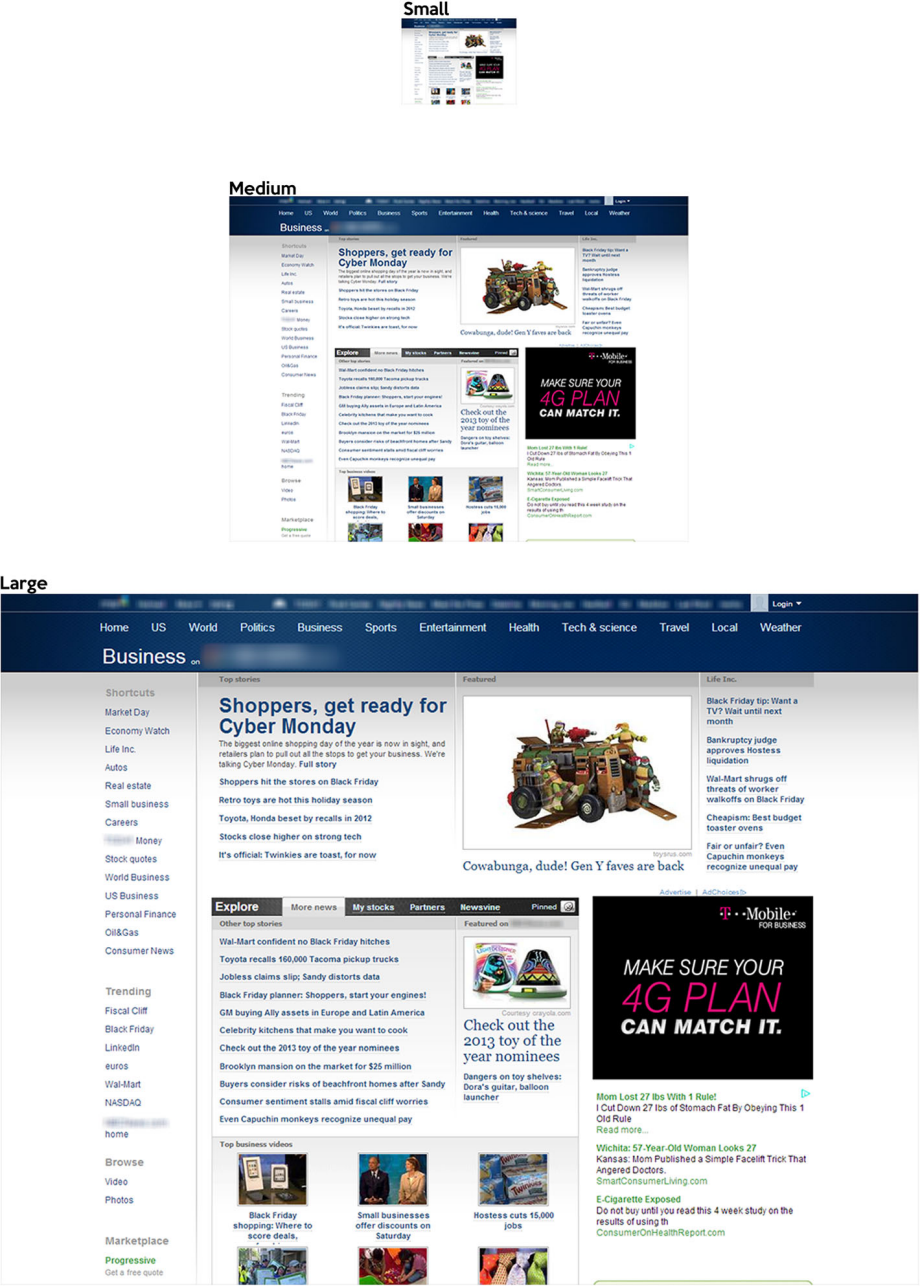
Relative size comparisons of the stimuli from Experiment 2

All visual stimuli were presented in three different sizes. At a viewing distance of 60 cm, these included 128 × 96 px (small, subtending 3.44° by 2.58°), 512 × 386 px (medium, subtending 13.69° by 10.34°), and 1024 × 772 px (large, subtending 27.01° by 20.52°) (Fig. 7).

**Fig. 8:**
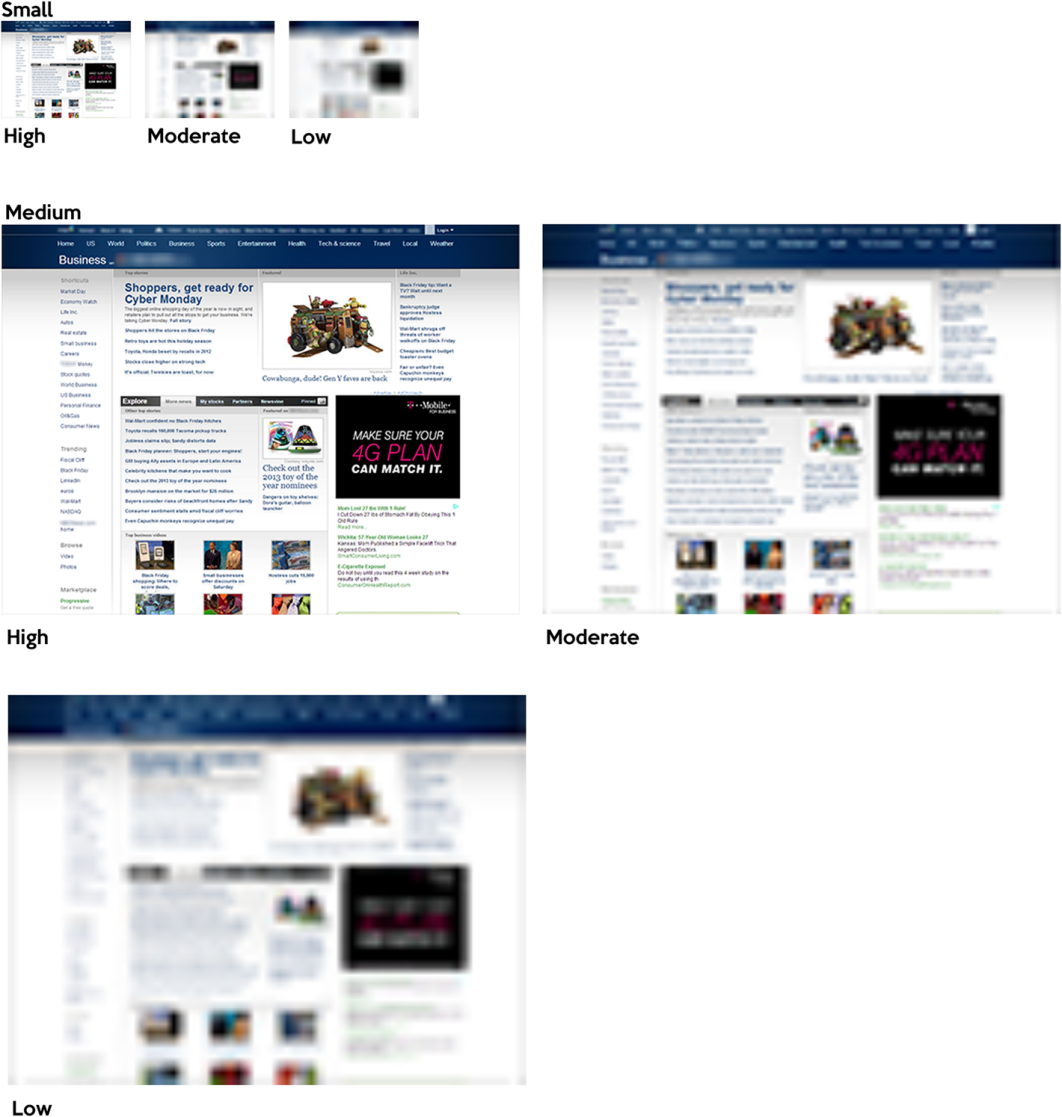
Comparison of size and resolution of small and medium images

**Fig. 9:**
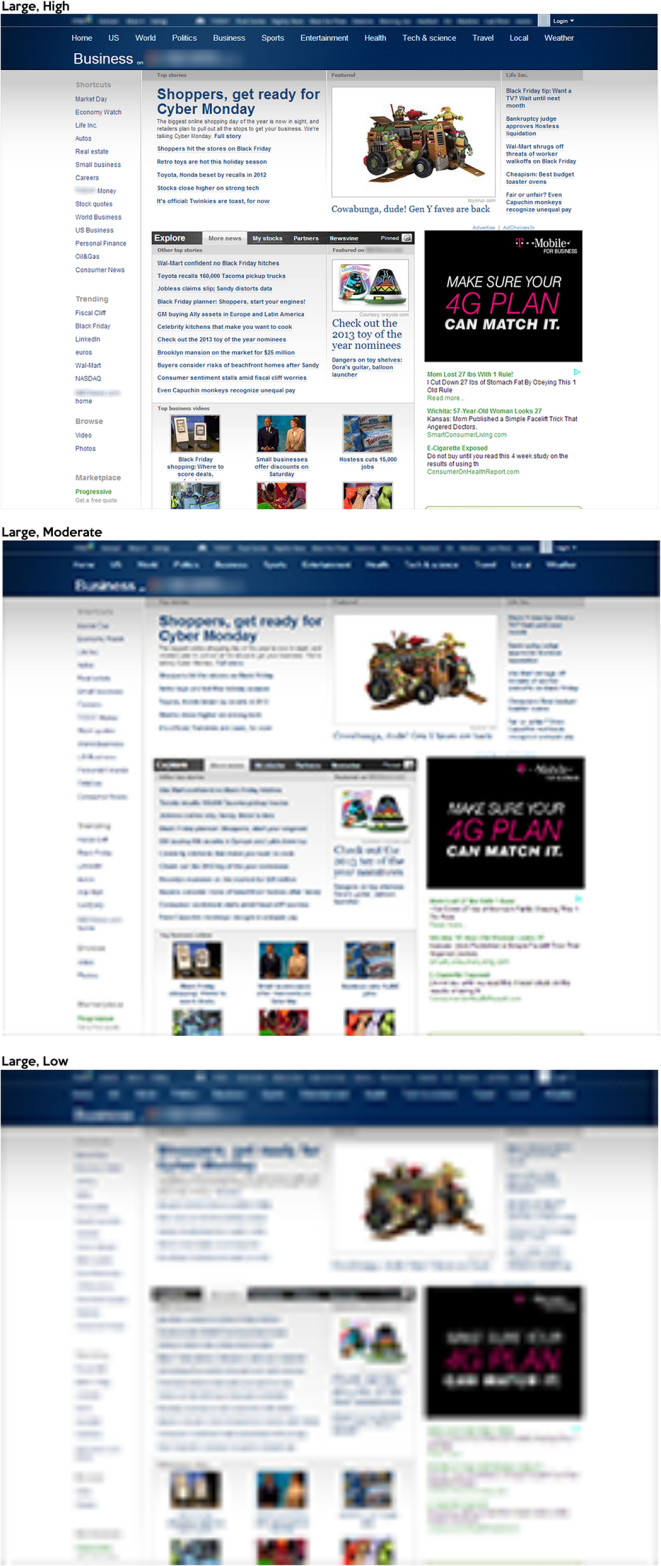
Comparison of size and resolution of large images

Image quality of the stimuli was manipulated using a procedure from Torralba (2009). The stimuli were displayed in three different resolutions, which included low, moderate, and high. High resolution stimuli were not subsampled to decrease resolution from the original resolution. In both the low and moderate resolution conditions, stimuli were sampled to sizes one-eighth and one-quarter of their original height and width dimensions. After the downsampling was completed, they were resized back up to their original dimensions. This effectively reduced the amount of visual information available in each resolution and provided an upper bound of the quantity of information available. For instance, in the high resolution condition, the small stimulus contained 128 × 96 px. At the moderate resolution, the small stimulus contained 32 × 24 px, but was displayed at 128 × 96 px (Figs. 8 and 9).

#### Procedure

**Fig. 10:**
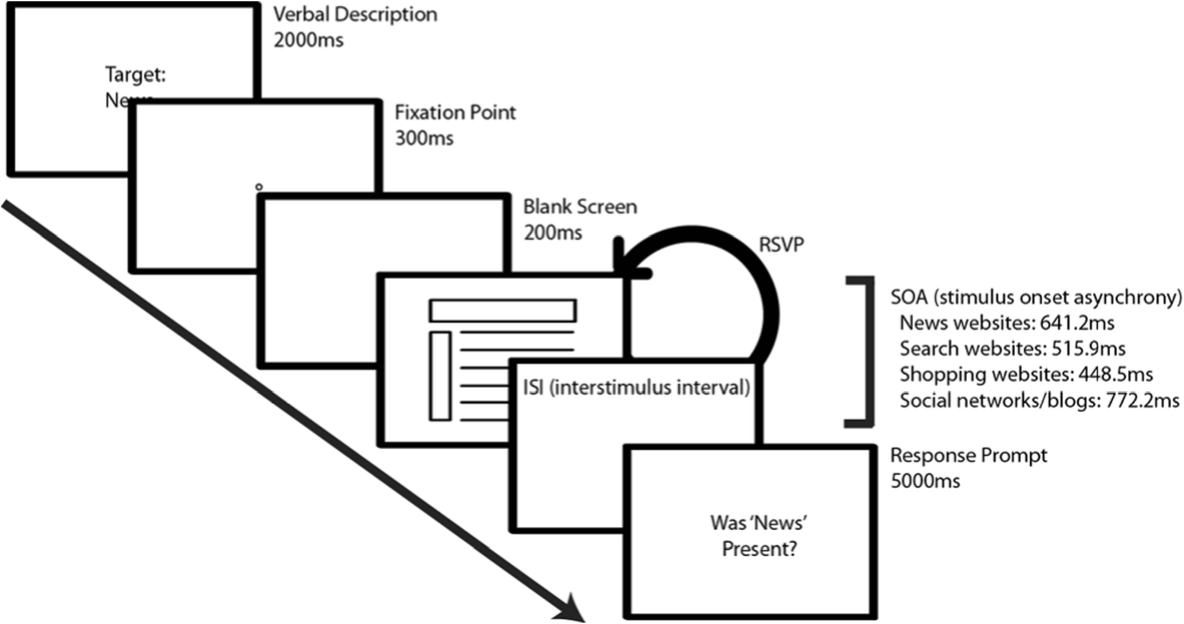
Sensitivity for detecting websites in each of the different size and resolutions. Error bars are within-subjects 95% confidence intervals (Cousineau, 2005; Morey, 2008)

In the experiment, participants completed 48 practice trials based on stimulus size, equating to 16 trials per condition. Each size/resolution combination had 16 experimental trials, equating to 192 trials per size or resolution, or 576 experimental trials overall. Half of the practice and experimental trials contained targets and half did not. Trials were organized into blocks by size; trials for target category and stimulus resolution were randomized across each block. Experiments were completed over two sessions. In the first session, users completed one block. In the second session, users completed two blocks. One- to two-minute breaks were provided every 15 min, and a longer break was given between the two blocks during the second session. Participants spent approximately 2 h completing each session. Finally, the presentation order of the blocks was counterbalanced to account for order effects. See Fig. 10 for a schematic of the trial.

### Results

#### A’ sensitivity measure

For each participant, sensitivity was determined by calculating A’ for the Yes/No response during the RSVP task. d’ has two assumptions that cannot be met or tested when using Yes/No tasks (Stanislaw & Todorov, 1999). Because using Yes/No with d’ violates these assumptions, Stanislaw & Todorov noted that non-parametric measures may be used instead. A’ was used as it is the most popular non-parametric measure of sensitivity.

Because A’ resembles a proportion and was bounded (0, 1), logit transformed values were used for analysis to meet parametric assumptions. Normality of the transformed data was checked across each combination of website type, stimulus size, and stimulus resolution. Overall, each resolution combined with the large search website category, and the low resolution, small size condition for the shopping website category, exhibited negative skewness, *p* < 0.01.

#### Sensitivity to targets by condition

**Fig. 11:**
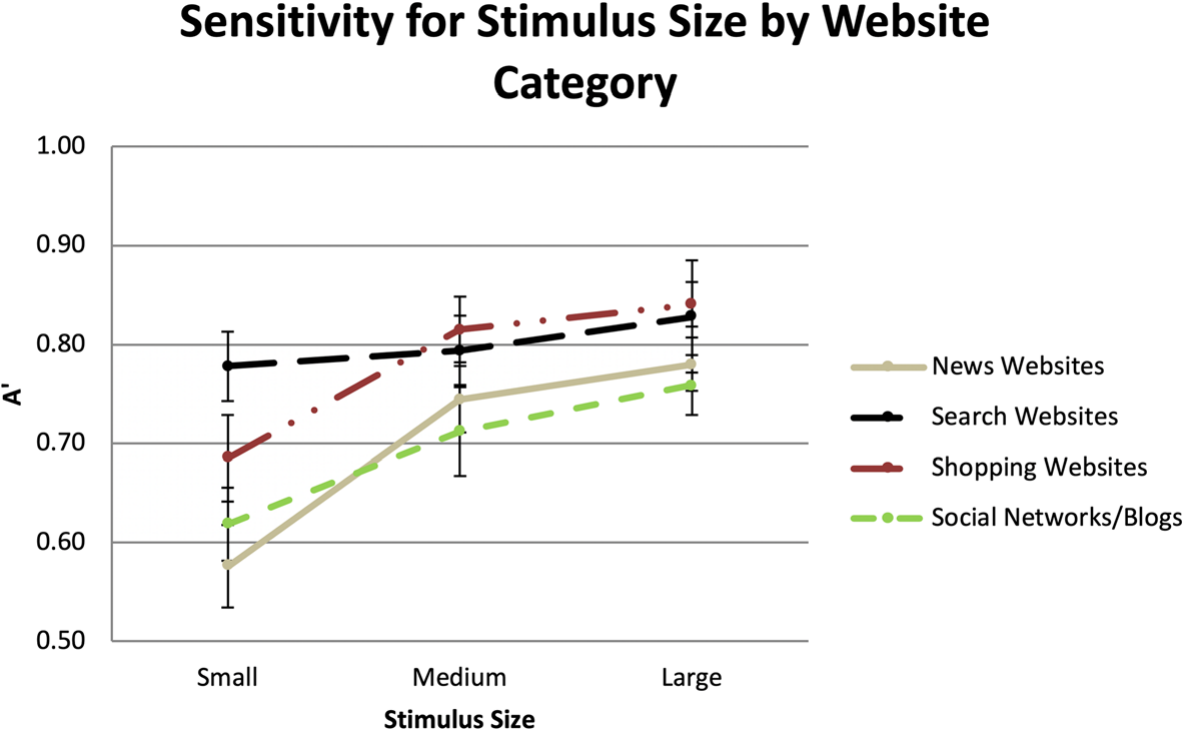
Sensitivity for detecting websites in each of the different sizes and resolutions. Error bars are within-subjects 95% confidence intervals (Cousineau, 2005; Morey, 2008)

The sensitivity to size and resolution conditions for each of the stimulus types were compared with the logit of 0.5, to indicate which conditions had targets that could not be distinguished from distractors during the RSVP task (Stanislaw & Todorov, 1999). The results of one-sample *t*-tests indicated that participants could not distinguish targets from distractors in both low and moderate resolutions in the small size condition for news websites, shopping websites, and social networks/blogs. Moreover, the same result was found for small size, high resolution news website condition. At medium and large sizes, regardless of resolution, participants had sensitivity indicating they were capable of distinguishing targets from the distractors. Finally, the results indicated that for search, participants had significantly higher sensitivity, regardless of size or resolution (Fig. 11).

#### Sensitivity to visual information vs resolution

The stimuli for the small size, high resolution, medium size, moderate resolution, and large size, low resolution all used the same source images (128 px), and thus contained a similar amount of visual information before being rendered in their final small, medium, and large sizes. The results of a repeated measures ANOVA indicated significant differences between the three conditions, *F*(6,120) = 6.28, *p* < 0.01, partial *η*^2^ = 0.24.

**Fig. 12:**
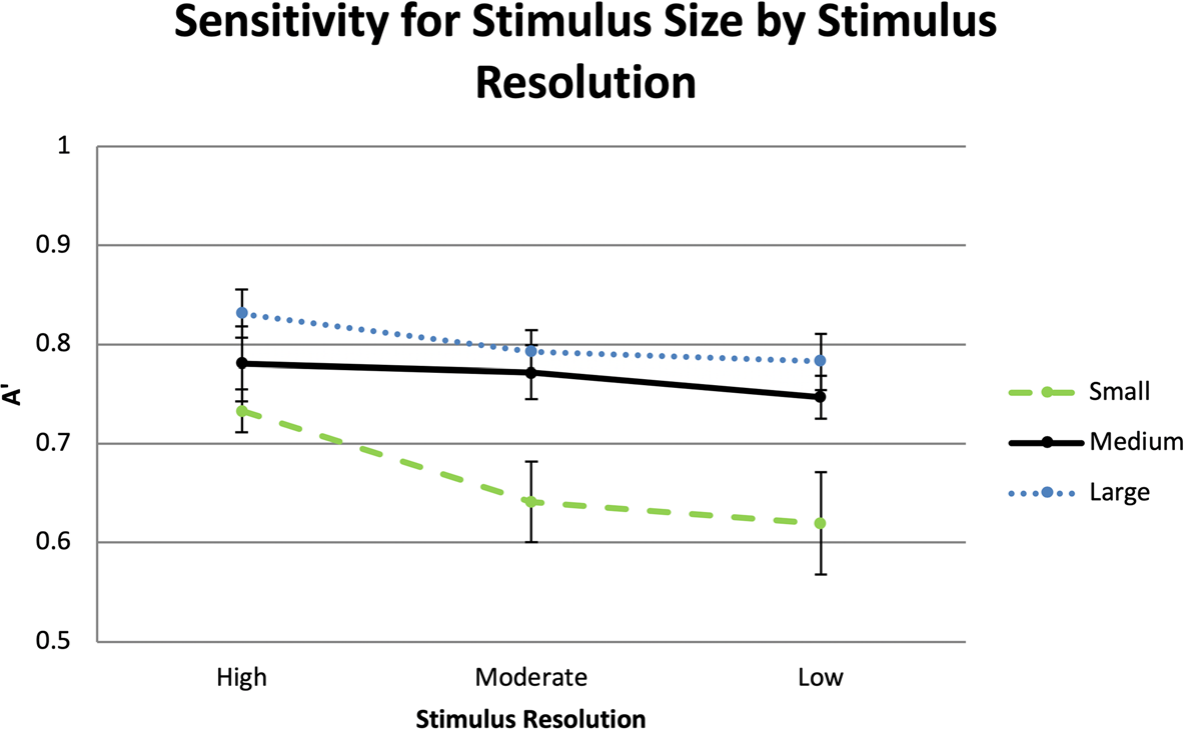
Sensitivity as a function of image size and resolution. Error bars are within-subjects 95% confidence intervals (Cousineau, 2005; Morey, 2008).

**Fig. 13:**
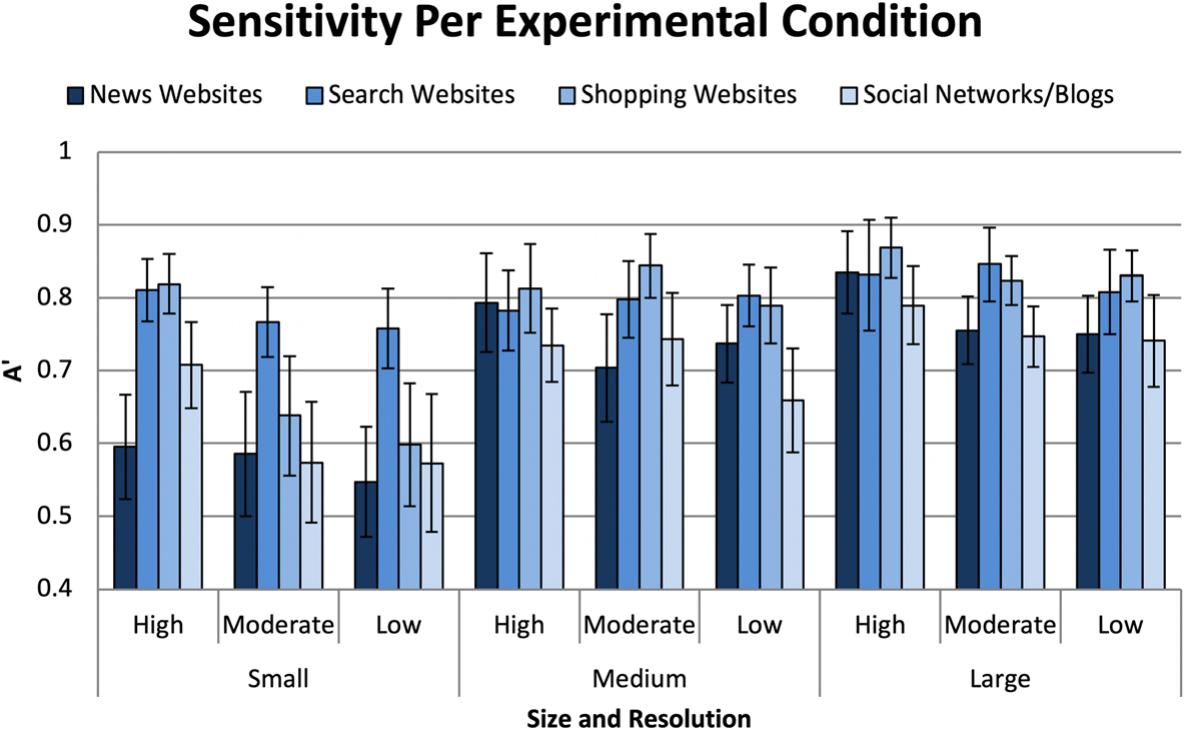
Sensitivity for detecting websites in each of the size and resolution conditions. Error bars are within-subjects 95% confidence intervals (Cousineau, 2005; Morey, 2008)

Planned comparisons detected no differences between the medium and the large size conditions, all *p* > 0.05. However, sensitivity was higher for large and medium sizes than the small size (*MD* = 0.05, *p* < 0.01; *MD* = 0.04, *p* < 0.01) (see Fig. 12 for interaction between size and resolution). This suggests that even though the resolution was higher for the small size, the increased size of the stimuli sufficiently mitigated decreases in resolution found in the larger sized stimuli (see Fig. 13 for the comparisons across all 36 conditions).

### Discussion

Overall, participants were able to detect websites across almost all experimental conditions. In conditions with the least visual information (small size, low and moderate resolutions), participants were unable to discern targets for three of the four types of websites (news websites, shopping websites, social networks/blogs) from distractors. However, even with these poor resolutions, participants could distinguish search websites from the other website categories. In the larger sizes, participants could detect targets with exposures of the stimulus not exceeding a fixation.

#### Stimulus properties aiding detection

Participants’ ability to detect search websites in the small and low-resolution conditions indicated that search websites may have distinguishing properties. Subjective comments by participants suggested that white space, blue text, advertising, and centered search bars were important for detecting search websites. In the small size, these features would still be distinct. In the lower resolutions of the small size, it seems probable that such features, or lack of pictures, helped distinguish search from the other types of websites.

In the lower resolution conditions of the small stimulus size, participants could not distinguish shopping, news, or social networks/blogs. In these conditions, the quality of web elements suffered from the decreased resolution. Given this, detection of these three types of websites appears to be dependent on web elements, such as pictures, headers, or other web elements. Indeed, participants indicated that images were an important discriminatory factor for discerning news websites, shopping websites, and social networks/blogs.

Oliva and Schyns (2000) found that color blobs provided sufficient context for participants to recognize various natural scenes. Torralba (2009) showed 8 × 8 px color images in the 256 px size and found above chance performance. In this study, visual information from a 32 × 24 px displayed in the small size (128 × 96 px) was insufficient for above chance performance, except for search websites. While there were task differences between the current study and Torralba (2009) (RSVP vs recognition tasks), these results suggest that the amount of visual information needed to detect low resolution websites is higher than the amount needed to detect natural scenes.

The relationship between size and resolution was not linear. When using the same source images at different sizes and resolutions, the benefit of image resolution can be mitigated by image size. This suggests that increasing the size of the local features may have made them more distinguishable, even though they were more degraded in resolution.

In summary, because users were able to detect websites from categorical prompts, this ability may transfer to situations in which users have a conceptual model for a website and thus may be able to quickly scan through thumbnails, provided the thumbnails were of a sufficient resolution and size to distinguish diagnostic elements such as pictures and headers. Consequently, these results have implications for visual bookmarks or the use of thumbnails in browser interfaces as a way of displaying frequently visited websites, which are found in browsers such as Google Chrome, Apple Safari, etc.

### Conclusions

The results of this study demonstrated that participants relied on local features to detect websites during the RSVP tasks in the study for shopping, news, and social network/blog websites but not search websites. When features become indistinguishable such that they cannot be processed meaningfully, sensitivity for detecting websites that depend on these features for identification decreases. The interaction between stimulus size and resolution demonstrated that even when resolution decreases, if image size is sufficient for meaningful processing of web elements, the decreases in resolution were not detrimental. When stimuli are small, decreases in resolution cause swift declines in performance.

## General discussion

These two experiments demonstrate that participants can recognize websites in a glance during an RSVP task, though not as quickly as natural scenes. Moreover, while participants may be relying on some gist-like representation of the websites in the detection task, they also seem to rely on diagnostic pictures to recognize website categories such as shopping and news. Thus, rapid website perception may utilize a combination of gist-like and diagnostic feature processing (also see Jahanian et al., 2018).

The SOAs required for website detection typically exceeded those for detecting upright and inverted natural scenes, which indicated that additional processing time was needed. These differences were generally meaningful, as indicated by the moderate and large effect sizes. However, the lack of significant differences and small effect sizes between shopping and search websites with natural scenes indicated more efficient performance for processing certain website categories. From discussions with participants, web elements may have aided detection. Participants stated they used specific web elements or diagnostic features to aid in the detection of websites in both Experiment 1 and 2, such as white space and blue text for search websites. The durations of the SOAs suggested that a few local features could likely be processed within this time. If these web elements were distinct or unique to the websites in question, detection of the website category could be based on detection of these elements or features in addition to or instead of global gist. A systematic exploration of the image properties—both global and local—that underlie the ability to detect web page stimuli in an RSVP stream is a topic worthy of future study.

The notion web elements are critical for perceiving websites in a glance was supported in the second study, where the size and resolution of websites were systematically manipulated. The results demonstrated that when the size of the stimulus decreased, sensitivity for detecting websites was diminished with the exception of search websites. Search websites typically lacked pictures and had other distinct visual attributes such as a high proportion of white and blue pixels that appear to have been resilient to decreases in stimulus size. However, for news, shopping, and social networks/blogs, decreases in stimulus size profoundly affected sensitivity, probably because the small stimulus sizes reduced the distinguishability of local features, such as pictures, text, or other web elements. This provided evidence that web elements were likely significant contributors to the detection of websites in this research. We performed several supplementary analyses of our stimulus set, including quantifying the average number of black, white, red, green, and blue pixels per website category and the average number of pictures of people and other objects per website category. The supplementary analyses and the original stimulus set may be downloaded from https://scholarworks.sjsu.edu/psych_pub/28/.

Overall, this research provides support for the idea that participants use local features or web elements, such as pictures and their content, for detecting news websites, shopping websites, and social networks/blogs. The duration of the SOAs for each type of website in this study would support the processing of a few local features, which was likely sufficient to detect targets during the RSVP streams. How search websites were detected was less clear. It is plausible that both web elements found on websites, or the global features of search websites, may have contributed to their detection.

It is interesting that participants could detect category targets of websites with above chance accuracy with such brief exposures. This strongly suggests that participants have schematic representations of websites for these particular genres. Additionally, this implies that such schemas are not necessarily unique to specific websites in the genre. For instance, if a user has a conceptual model of a shopping website, their model would be applicable to multiple shopping websites, like eBay or Amazon, instead of only a specific shopping website.

This research also suggests that conceptual information about websites can be extracted from small screenshots with, in some cases, subpar resolution. These findings are directly applicable to interfaces that use thumbnails of websites, such as smartphones or web browsers that display favorite or frequently visited websites. The SOAs, presentation times, stimulus sizes, and stimulus resolutions provide some guidance about the image quality and display rate necessary to facilitate rapid scanning of thumbnails and how variations may affect user performance.

Our results suggest that both global and local aspects of websites need to be taken into consideration during product development life cycles. This research, in conjunction with previous research on first impressions of websites, illustrates that a multitude of information, such as the quality of the website and other semantic information, can be perceived from websites within a glance. This information ranges from global characteristics of web pages (e.g., visual appeal) to specific elements found on web pages (e.g., pictures). Research has shown that such quick judgments are stable for extended viewing durations and it may be posited that extracted semantic information can influence user behavior during extended interaction. Designers and developers may need to consider perception and conceptualizations of websites from the first glance to extended periods of interaction. If designers and developers ignore early perceptual and conceptual aspects of websites, they may be handicapping the usability and interaction of the website in later interactions by ignoring information gleaned much earlier.

## Data Availability

The datasets used and/or analyzed during the current study are available from the corresponding author on reasonable request. The supplementary analyses and entire stimulus dataset can be downloaded from https://scholarworks.sjsu.edu/psych_pub/28/.

## Abbreviations

ANOVA: Analysis of variance
ISI: Inter-stimulus interval
RSVP: Rapid serial visual presentation
SOA: Stimulus onset asynchrony

## References

[CR1] Albert W, Gribbons W, Almadas J (2009). Pre-conscious assessment of trust: A case study of financial and health care web sites. Proceedings of the Human Factors and Ergonomics Society Annual Meeting.

[CR2] Benway JP (1998). Banner blindness: The irony of attention grabbing on the world wide web. Proceedings of the Human Factors and Ergonomics Society 42nd Annual Meeting.

[CR3] Bernard M, Sheshadri A (2004). Preliminary examination of global expectations of users’ mental models for e-commerce web layouts. Usability News.

[CR4] Bernard ML (2001). Developing schemas for the location of common web objects. Proceedings of the Human Factors and Ergonomics Society Annual Meeting.

[CR5] Bernard ML (2003). Examining user expectations for the location of common e-commerce web objects. Proceedings of the Human Factors and Ergonomics Society Annual Meeting.

[CR6] Biederman I, Mezzanotte RJ, Rabinowitz JC (1982). Scene perception: Detecting and judging objects undergoing relational violations. Cognitive Psychology.

[CR7] Brainard DH (1997). The psychophysics toolbox. Spatial Vision.

[CR8] Chittaro L (2006). Visualizing information on mobile devices. Computer.

[CR9] Cousineau D (2005). Confidence intervals in within-subjects designs: A simpler solution to Loftus and Masson’s method. Tutorial in Quantitative Methods for Psychology.

[CR10] Crowston K, Williams M (2000). Reproduced and emergent genres of communication on the World Wide Web. The Information Society.

[CR11] Davenport JL (2007). Consistency effects between objects in scenes. Memory & Cognition.

[CR12] Davenport JL, Potter MC (2004). Scene consistency in object and background perception. Psychological Science.

[CR13] Di Nocera F, Capponi C, Ferlazzo F (2004). Finding geometrical associations between meaningful objects in the web: A geostatistical approach. PsychNology Journal.

[CR14] Diamond R, Carey S (1986). Why faces are and are not special: An effect of expertise. Journal of Experimental Psychology: General.

[CR15] Dillon A, Gushrowski BA (2000). Genres and the Web: Is the personal home page the first uniquely digital genre?. Journal of the American Society for Information Science.

[CR16] Epstein, R. A., Higgins, J. S., Parker, W., Aguirre, G. K., & Cooperman, S. (2006). Cortical correlates of face and scene inversion: A comparison. *Neuropsychologia*, *44*(7), 1145–1158. 10.1016/j.neuropsychologia.2005.10.009 16303149

[CR17] Evans KK, Treisman A (2005). Perception of objects in natural scenes: Is it really attention free?. Journal of Experimental Psychology-Human Perception and Performance.

[CR18] Fei-Fei L, Iyer A, Koch C, Perona P (2007). What do we perceive in a glance of a real-world scene?. Journal of Vision.

[CR19] Fei-Fei L, VanRullen R, Koch C, Perona P (2002). Rapid natural scene categorization in the near absence of attention. Proceedings of the National Academy of Sciences.

[CR20] Friedman A (1979). Framing pictures: The role of knowledge in automatized encoding and memory for gist. Journal of Experimental Psychology: General.

[CR21] Granka, L., Hembrooke, H., & Gay, G. (2006). Location location location: Viewing patterns on WWW pages. In *Proceedings of the 2006 Symposium on Eye Tracking Research & Applications*, (p. 43). New York: ACM. 10.1145/1117309.1117328.

[CR22] Greene MR, Oliva A (2009). The briefest of glances: The time course of natural scene understanding. Psychological Science.

[CR23] Greene MR, Oliva A (2009). Recognition of natural scenes from global properties: Seeing the forest without representing the trees. Cognitive Psychology.

[CR24] Harding G, Bloj M (2010). Real and predicted influence of image manipulations on eye movements during scene recognition. Journal of Vision.

[CR25] Henderson JM, Hollingworth A (1999). High-level scene perception. Annual Review of Psychology.

[CR26] Intraub H (1980). Presentation rate and the representation of briefly glimpsed pictures in memory. Journal of Experimental Psychology: Human Learning and Memory.

[CR27] Intraub H (1981). Rapid conceptual identification of sequentially presented pictures. Journal of Experimental Psychology: Human Perception and Performance.

[CR28] Intraub H (1984). Conceptual masking: The effects of subsequent visual events on memory for pictures. Journal of Experimental Psychology: Learning, Memory, and Cognition.

[CR29] Jahanian A, Keshvari S, Rosenholtz R (2018). Web pages: What can you see in a single fixation?. Cognitive Research: Principles and Implications.

[CR30] Jiang Z, Wang W, Tan BC, Yu J (2016). The determinants and impacts of aesthetics in users’ first interaction with websites. Journal of Management Information Systems.

[CR31] Joubert OR, Rousselet GA, Fabre-Thorpe M, Fize D (2009). Rapid visual categorization of natural scene contexts with equalized amplitude spectrum and increasing phase noise. Journal of Vision.

[CR32] Kelley TA, Chun MM, Chua KP (2003). Effects of scene inversion on change detection of targets matched for visual salience. Journal of Vision.

[CR33] Kirchner H, Thorpe SJ (2006). Ultra-rapid object detection with saccadic eye movements: Visual processing speed revisited. Vision Research.

[CR34] Kleiner, M., Brainard, D., & Pelli, D. (2007). What’s New in Psychtoolbox-3? Perception [Online], 36, ECVP Abstract Supplement. https://pure.mpg.de/rest/items/item_1790332/component/file_3136265/content.

[CR35] Knoche, H., McCarthy, J. D., & Sasse, M. A. (2005). Can small be beautiful? Assessing image resolution requirements for mobile TV. In *Proceedings of the 13th Annual ACM International Conference on Multimedia*, (pp. 829–838). New York: ACM. 10.1145/1101149.1101331.

[CR36] Lam, H., & Baudisch, P. (2005). Summary thumbnails: Readable overviews for small screen web browsers. In *Proceedings of the SIGCHI Conference on Human factors in Computing Systems*, (pp. 681–690). New York: ACM. 10.1145/1054972.1055066.

[CR37] Larson AM, Loschky LC (2009). The contributions of central versus peripheral vision to scene gist recognition. Journal of Vision.

[CR38] Lindgaard G, Dudek C, Sen D, Sumegi L, Noonan P (2011). An exploration of relations between visual appeal, trustworthiness and perceived usability of homepages. ACM Transactions on Computer-Human Interaction (TOCHI).

[CR39] Lindgaard G, Fernandes G, Dudek C, Brown J (2006). Attention web designers: You have 50 milliseconds to make a good first impression!. Behaviour & Information Technology.

[CR40] Liu H, Agam Y, Madsen JR, Kreiman G (2009). Timing, timing, timing: Fast decoding of object information from intracranial field potentials in human visual cortex. Neuron.

[CR41] Loftus GR, Nelson WW, Kallman HJ (1983). Differential acquisition rates for different types of information from pictures. The Quarterly Journal of Experimental Psychology.

[CR42] Loftus GR, Shimamura AP, Johnson CA (1985). How much is an icon worth?. Journal of Experimental Psychology: Human Perception and Performance.

[CR43] Loschky LC, Hansen BC, Sethi A, Pydimarri TN (2010). The role of higher order image statistics in masking scene gist recognition. Attention, Perception, & Psychophysics.

[CR44] Loschky LC, Larson AM (2010). The natural/man-made distinction is made before basic-level distinctions in scene gist processing. Visual Cognition.

[CR45] Maniar N, Bennett E, Hand S, Allan G (2008). The effect of mobile phone screen size on video based learning. Journal of Software.

[CR46] McCarthy, J. D., Sasse, M. A., & Riegelsberger, J. (2004). Could I have the menu please? An eye tracking study of design conventions. In People and computers XVII—Designing for society (pp. 401–414). Springer, London.

[CR47] Meng M, Potter MC (2008). Detecting and remembering pictures with and without visual noise. Journal of Vision.

[CR48] Morey RD (2008). Confidence intervals from normalized data: A correction to Cousineau (2005). Tutorial in Quantitative Methods for Psychology.

[CR49] Nandakumar C, Malik J (2009). Understanding rapid category detection via multiply degraded images. Journal of Vision.

[CR50] Navon D (1977). Forest before trees: The precedence of global features in visual perception. Cognitive Psychology.

[CR51] Neisser U (1967). Cognitive psychology.

[CR52] Nielsen (2012). State of the media: U.S. digital consumer report, Q3-Q4 2011.

[CR53] Oliva A (2005). Gist of the scene. Neurobiology of Attention.

[CR54] Oliva A, Schyns PG (1997). Coarse blogs or fine edges? Evidence that information diagnosticity change the perception of complex visual stimuli. Cognitive Psychology.

[CR55] Oliva, A., & Schyns, P. G. (2000). Diagnostic Colors Mediate Scene Recognition. Cognitive Psychology *41*(2), 176–210. 10.1006/cogp.1999.0728 10968925

[CR56] Oliva A, Torralba A (2001). Modeling the shape of the scene: A holistic representation of the spatial envelope. International Journal of Computer Vision.

[CR57] Oliva A, Torralba A (2006). Building the gist of a scene: The role of global image features in recognition. Progress in Brain Research.

[CR58] Oliva A, Torralba A (2007). The role of context in object recognition. Trends in Cognitive Science.

[CR59] Owens JW, Chaparro BS, Palmer EM (2011). Text advertising blindness: the new banner blindness?. Journal of Usability Studies.

[CR60] Owens JW, Palmer EM, Chaparro BS (2014). The pervasiveness of text advertising blindness. Journal of Usability Studies.

[CR61] Pelli DG (1987). The ideal psychometric procedure. Investigative Ophthalmology & Visual Science.

[CR62] Pelli DG (1997). The VideoToolbox software for visual psychophysics: Transforming numbers into movies. Spatial Vision.

[CR63] Pew Research (2013). http://www.pewresearch.org/fact-tank/2016/09/07/some-americans-dont-use-the-internet-who-are-they/

[CR64] Potter MC (1975). Meaning in visual search. Science.

[CR65] Potter MC (1976). Short-term conceptual memory for pictures. Journal of Experimental Psychology: Human Learning and Memory.

[CR66] Potter MC, Fox LF (2009). Detecting and remembering simultaneous pictures in a rapid serial visual presentation. Journal of Experimental Psychology: Human Perception and Performance.

[CR67] Rayner K (2009). The 35th Sir Frederick Bartlett Lecture: Eye movements and attention in reading, scene perception, and visual search. Quarterly Journal of Experimental Psychology.

[CR68] Rehm, G. (2002). Towards automatic web genre identification: A corpus-based approach in the domain of academia by example of the Academic’s Personal Homepage. In *Proceedings of the 35th Annual Hawaii International Conference on System Sciences*, (pp. 1143–1152). New York: IEEE. 10.1109/HICSS.2002.994036.

[CR69] Rosch E, Rosch E, Lloyd B (1978). Principles of categorization. Cognition and categorization.

[CR70] Roth S, Schmutz P, Pauwels S, Bargas-Avila J, Opwis K (2010). Mental models for web objects: Where do users expect to find the most frequent objects in online shops, news portals, and company web pages?. Interacting with Computers.

[CR71] Rousselet, G. A., Macé, M. J. M., & Fabre-Thorpe, M. (2003). Is it an animal? Is it a human face? Fast processing in upright and inverted natural scenes. Journal of vision, *3*(6), 5–5. 10.1167/3.6.5 12901715

[CR72] Rousselet GA, Joubert OR, Fabre-Thorpe M (2005). How long to get to the “gist” of real-world natural scenes. Visual Cognition.

[CR73] Ryan, T., Field, R. H., & Olfman, L. (2002). Homepage genre dimensionality. In *Proceedings of the Eighth Americas Conference on Information Systems.*, (pp. 1116–1128). Association for Information Systems, Dallas.

[CR74] Santa-Maria, L., & Dyson, M. C. (2008). The effect of violating visual conventions of a website on user performance and disorientation: How bad can it be? In *Proceedings of the 26th annual ACM International Conference on Design of Communication*, (pp. 47–54). New York: ACM. 10.1145/1456536.1456547.

[CR75] Santini, M. (2006). Identifying genres of web pages. In *Proceedings of TALN 2006*, (pp. 307–316). Louvain-la-Neuve: UCL Press.

[CR76] Santini, M. (2007). Characterizing genres of web pages: Genre hybridism and individualization. In *Proceedings of the 40th Annual Hawaii International Conference on System Sciences*, (pp. 71–71). New York: IEEE. 10.1109/HICSS.2007.124.

[CR77] Shaikh AD, Chaparro BS, Joshi A (2006). Indian users’ expectations for the location of web objects on informational websites. Proceedings of the Human Factors and Ergonomics Society Annual Meeting.

[CR78] Shaikh AD, Lenz K (2006). Where’s the search? Re-examining user expectations of web objects. Usability News.

[CR79] Shore DI, Klein RM (2000). The effects of scene inversion on change blindness. The Journal of General Psychology.

[CR80] Sperling G (1960). The information available in brief visual presentations. Psychological Monographs: General and Applied.

[CR81] Stanislaw H, Todorov N (1999). Calculation of signal detection theory measures. Behavior Research Methods, Instruments, & Computers.

[CR82] Thielsch MT, Hirschfeld G (2012). Spatial frequencies in aesthetic website evaluations – explaining how ultra-rapid evaluations are formed. Ergonomics.

[CR83] Torralba A (2009). How many pixels make an image. Visual Neuroscience.

[CR84] Torralba A, Oliva A, Castelhano MS, Henderson JM (2006). Contextual guidance of eye movements and attention in real-world scenes: The role of global features on object search. Psychological Review.

[CR85] Tuch AN, Presslaber EE, Stocklin M, Opwis K, Bargas-Aliva JA (2012). The role of visual complexity and prototypicality regarding first impression of websites: Working towards understanding aesthetic judgments. International Journal of Human-Computer Studies.

[CR86] Turk-Browne NB, Jungé JA, Scholl BJ (2005). The automaticity of visual statistical learning. Journal of Experimental Psychology: General.

[CR87] Tzanidou Ekaterini, Petre Marian, Minocha Shailey, Grayson Andrew (2005). Combining Eye Tracking and Conventional Techniques for Indications of User-Adaptability. Human-Computer Interaction - INTERACT 2005.

[CR88] Watson AB, Pelli DG (1983). QUEST: a Bayesian adaptive psychometric method. Perception & Psychophysics.

[CR89] Wolfe JM, Võ ML, Evans KK, Greene MR (2011). Visual search in scenes involves selective and nonselective pathways. Trends in Cognitive Sciences.

[CR90] Xiao, J., Hays, J., Ehinger, K. A., Oliva, A., & Torralba, A. (2010). Sun database: Large-scale scene recognition from abbey to zoo. In *2010 IEEE Conference on Computer Vision and Pattern Recognition (CVPR)*, (pp. 3485–3492). New York: IEEE. 10.1109/CVPR.2010.5539970.

[CR91] Zimmermann E, Schnier F, Lappe M (2010). The contribution of scene context on change detection performance. Vision Research.

